# Specific detection of high mobility group box 1 degradation product with a novel ELISA

**DOI:** 10.1186/s10020-021-00323-1

**Published:** 2021-06-09

**Authors:** Takaaki Totoki, Takashi Ito, Shingo Yamada, Goichi Honda, Tsuyoshi Hattori, Ikuro Maruyama

**Affiliations:** 1 Department of Systems Biology in Thromboregulation, Kagoshima University Graduate School of Medical and Dental Sciences, 8-35-1 Sakuragaoka, Kagoshima, 890-8544 Japan; 2 R&D Center, Shino-Test Corporation, Sagamihara, Japan; 3 Department of Medical Affairs, Asahi Kasei Pharma Corporation, Tokyo, Japan

## Abstract

**Background:**

During sepsis or sterile tissue injury, the nuclear protein high mobility group box 1 (HMGB1) can be released to the extracellular space and ultimately into systemic circulation, where it mediates systemic inflammation and remote organ failure. The proinflammatory effects of HMGB1 can be suppressed by recombinant thrombomodulin (rTM), in part through a mechanism involving thrombin–rTM-mediated degradation of HMGB1. Given that HMGB1 is proinflammatory but the HMGB1 degradation product (desHMGB1) is not, an analytical method that discriminates between these two molecules may provide a more in-depth understanding of HMGB1-induced pathogenicity as well as rTM-mediated therapeutic efficiency.

**Methods:**

A peptide that has a shared amino-terminal structure with desHMGB1 was synthesized. C3H/*lpr* mice were immunized with the desHMGB1 peptide conjugate, and antibody-secreting hybridoma cells were developed using conventional methods. The reactivity and specificity of the antibodies were then analyzed using antigen-coated enzyme-linked immunosorbent assay (ELISA) as well as antibody-coated ELISA. Next, plasma desHMGB1 levels were examined in a cecal ligation and puncture (CLP)-induced septic mouse model treated with rTM.

**Results:**

Through a series of screening steps, we obtained a monoclonal antibody that recognized desHMGB1 but did not recognize intact HMGB1. ELISA using this antibody specifically detected desHMGB1, which was significantly increased in CLP-induced septic mice treated with rTM compared with those treated with saline.

**Conclusions:**

In this study, we obtained a desHMGB1-specific monoclonal antibody. ELISA using the novel monoclonal antibody may be an option for the in-depth analysis of HMGB1-induced pathogenicity as well as rTM-mediated therapeutic efficiency.

**Supplementary Information:**

The online version contains supplementary material available at 10.1186/s10020-021-00323-1.

## Background

Sepsis, which is life-threatening organ dysfunction caused by a dysregulated host response to infection, is a leading cause of mortality and critical illness worldwide (Singer et al. 2016). Although the details of the dysregulated host response during sepsis have not yet been clarified, previous studies have suggested that high mobility group box 1 protein (HMGB1) may play a significant role in sepsis-induced lethality (Wang et al. 1999, 2001). HMGB1 is a nuclear protein that binds to the minor groove of DNA without sequence specificity; it modulates gene transcription in almost all eukaryotic cells. In addition to its intracellular roles, HMGB1 has distinct functions in the extracellular milieu when released from damaged or activated cells. Extracellular HMGB1 acts on pattern recognition receptors, such as Toll-like receptors and receptor for advanced glycation end-products, and promotes inflammation, immune responses, epithelial barrier dysfunction, and even death if it accumulates excessively (Lotze and Tracey 2005; Andersson and Tracey 2011). Neutralizing antibodies against HMGB1 reverse the lethality of established sepsis in mice, indicating that HMGB1 may be a therapeutic target for sepsis with a clinically relevant therapeutic window (Yang et al. 2004).

Acting as a signal of tissue damage, extracellular HMGB1 is involved in the pathogenesis of sterile inflammatory diseases as well as infectious diseases. Extracellular HMGB1 levels are increased in ischemia–reperfusion injury (Andrassy et al. 2008; Yamamoto et al. 2010), heatstroke (Tong et al. 2011), acute coronary syndrome (Hashimoto et al. 2012), and rheumatoid arthritis (Taniguchi et al. 2003), and are associated with poor outcomes (Hatada et al. 2005; Volz et al. 2012). These findings suggest the importance of analyzing HMGB1 levels in inflammatory diseases; however, a mixture of different HMGB1 subtypes is present in analytical samples, which poses a challenge for the in-depth understanding of HMGB1-induced pathogenicity (Xue et al. 2021).

Thrombomodulin is an anticoagulant protein that is expressed mainly on the surface of endothelial cells (Ito et al. 2016). Recombinant thrombomodulin (rTM, also known as ART-123) is currently used in Japan for the treatment of disseminated intravascular coagulation (DIC), and is also a late-stage drug candidate for the treatment of severe sepsis with coagulopathy worldwide (Saito et al. 2007; Vincent et al. 2019; François et al. 2021). We have previously demonstrated that the proinflammatory effects of HMGB1 can be suppressed by rTM (Abeyama et al. 2005), in part through a mechanism involving thrombin–rTM-mediated degradation of HMGB1 (Ito et al. 2008). This yields a small fragment that consists of the amino-terminal domain (NTD) of HMGB1 and a large fragment that begins from Gly11 of HMGB1 (des-NTD HMGB1, or desHMGB1). Given that HMGB1 is proinflammatory but desHMGB1 is not, an analytical method that allows the accurate discrimination of these two molecules may provide a more in-depth understanding of HMGB1-induced pathogenicity, as well as of rTM-mediated therapeutic efficiency. In the present study, we screened for monoclonal antibodies that recognize desHMGB1 but do not recognize intact HMGB1 for the discriminative analysis of HMGB1 subtypes.

## Methods

### HMGB1 and desHMGB1

HMGB1 was purified from porcine thymus using the method reported by Sanders (1977). desHMGB1 was prepared by incubating purified HMGB1 with 20 U/mL of thrombin (Mochida Pharmaceutical) at 37 °C (Ito et al. 2008), and was then purified by ion-exchange chromatography. The purity of HMGB1 and desHMGB1 was confirmed by sodium dodecyl sulfate-polyacrylamide gel electrophoresis (SDS-PAGE) followed by Coomassie Brilliant Blue staining.

### Synthetic peptides for immunization and enzyme-linked immunosorbent assay (ELISA)

The peptides corresponding to Lys7–Ala17 (KKPRGKMSSYA) and Gly11–Ala17 (GKMSSYA) of HMGB1 were synthesized using a peptide synthesizer 430A (Applied Biosystems). Cysteine residues were then added to the carboxy-terminal tails of the peptides to allow directional conjugation to carrier proteins. After purification with high-performance liquid chromatography, the peptides were conjugated to carrier proteins (either bovine serum albumin (BSA) or bovine thyroglobulin) using an m-maleimidobenzoyl-*N*-hydroxysuccinimide ester crosslinker (Thermo Fisher Scientific) according to the manufacturer’s instructions. The conjugates were dialyzed against water and then freeze-dried. The peptides corresponding to Lys7–Gln21 (KKPRGKMSSYAFFVQ) and Gly11–Gln21 (GKMSSYAFFVQ) of HMGB1, followed by the sequence recognized by the capture antibody in the HMGB1 ELISA kit, were also synthesized. They were named the N-peptide and P-peptide, respectively.

### Preparation and characterization of monoclonal antibodies against desHMGB1

Monoclonal antibodies that selectively recognized desHMGB1 were prepared by Immuno-Biological Laboratories. Briefly, C3H/*lpr* mice were immunized five times with 50 μg of the desHMGB1 peptide conjugate (GKMSSYA-C-thyroglobulin). Serum samples were then obtained from four immunized mice, and the reactivity to desHMGB1 was assessed using ELISA. Spleen cells and lymph node cells were isolated from the immunized donor whose serum showed the highest reactivity to desHMGB1, and these cells were fused with X63 mouse myeloma cells using the method reported by Milstein (KÖHler and Milstein 1975). The hybridoma population was then distributed into 3800 wells, and the supernatants from each well were tested for their reactivity to the desHMGB1 peptide conjugate (GKMSSYA-C-BSA). Of these, the 95 supernatants with the high reactivity to desHMGB1 underwent the second screening, where those that were reactive to the P-peptide but unreactive to the N-peptide were selected. Three hybridoma populations with promising supernatants were then distributed into 95 wells each, and the same screening procedure was again conducted to obtain a single hybridoma clone that produced an antibody that selectively recognized desHMGB1. Anti-desHMGB1 IgG was then purified using protein A.

### Western blot analysis

Purified HMGB1 and desHMGB1 were subjected to SDS-PAGE with 5–20% or 10–20% acrylamide gradient gels (ATTO Corporation), transferred to nitrocellulose membranes (GE Healthcare), blocked with Block Ace (DS Pharma Biomedical), and incubated with rabbit anti-HMGB1 polyclonal antibody (18256; Abcam) or mouse anti-desHMGB1 monoclonal antibody at 4 °C overnight. Next, the membranes were washed with Tris-buffered saline containing 0.02% Tween-20, and then incubated with horseradish peroxidase-conjugated secondary antibodies (GE Healthcare) followed by detection with the Immobilon Western Chemiluminescent detection system (Merck Millipore).

### ELISA for desHMGB1

Polystyrene microtiter plates (Nunc) were coated with 100 μL/well of the capture antibody against HMGB1 (Shino-Test Corporation) in phosphate-buffered saline (PBS), and incubated overnight at 37 ℃. After three washes with PBS containing 0.05% Tween-20, the remaining binding sites were blocked by incubation with 400 μL/well of PBS containing 1% BSA. The plates were then washed again before being incubated with 100 μL/well of diluted standard and plasma samples (1:5 dilution in 0.2 mol/L Tris pH 6.5, 0.15 mol/L NaCl, and 1% BSA) for 24 h at room temperature. After washing, the plates were incubated with 100 μL/well of anti-desHMGB1 peroxidase-conjugated antibody for 2 h at room temperature. The plates were washed again, and were then incubated with the chromogenic substrate 3,3ʹ,5,5ʹ-tetra-methylbenzidine (Dojindo Laboratories) for 30 min at room temperature. The reaction was terminated by the addition of 0.35 mol/L of Na_2_SO_4_, and the optical density of each well was analyzed using a microplate reader (Bio-Rad Laboratories) set to 450 nm. The amino acid sequence of HMGB1 is highly conserved throughout species, and the sequence of the antibody recognition site used in this ELISA completely matched between humans and mice.

### Cecal ligation and puncture (CLP) in mice

Experiments involving animals were approved by the Institutional Animal Care and Use Committee of Kagoshima University, Kagoshima, Japan, and complied with the ARRIVE guidelines. Male C57BL/6J mice (Kyudo) were anesthetized with isoflurane at 10 weeks of age and subjected to CLP-induced sepsis as described previously (Rittirsch et al. 2009), with slight modifications. Briefly, the cecum of each volatile-anesthetized mouse was ligated at a site 1.5 cm distant from the tip of the cecum. For a mild model of sepsis, the tip of the cecum was punctured once with a 25-gauge needle. For a severe model of sepsis, the midway point between the ligation and the tip of the cecum was penetrated by a single through-and-through puncture with a 25-gauge needle. The cecum was returned to the abdominal cavity, and the abdominal wall was sutured. In some experiments, 2 mg/kg of HMGB1, desHMGB1, or saline was administered into the tail vein at 6 and 24 h after CLP, and the survival rate was monitored for 7 days. The general condition of the mice was evaluated every 6 h, and the mice were sacrificed when moribund. In another set of experiments, 5 mg/kg of rTM (Asahi Kasei Pharma) was administered into the tail vein at 6 h after CLP. Blood samples were then collected from volatile-anesthetized mice at 0, 6, 12, and 24 h after CLP, and were anticoagulated with one-tenth volume of 3.2% sodium citrate and centrifuged at 1500×*g* for 10 min. Plasma samples were stored at − 80 °C until further analysis.

### Statistical analyses

All statistical analyses were performed using GraphPad Prism v8 (GraphPad Software). Data are presented as means ± standard deviations. Statistical significance was determined using the Holm–Šidák method, with alpha = 0.05. For the comparison of survival between groups, the Kaplan–Meier method and the log-rank (Mantel–Cox) test were used. The sample size was estimated based on the available data regarding the effects of HMGB1, desHMGB1, and rTM on CLP-induced sepsis.

## Results

**Fig. 1:**
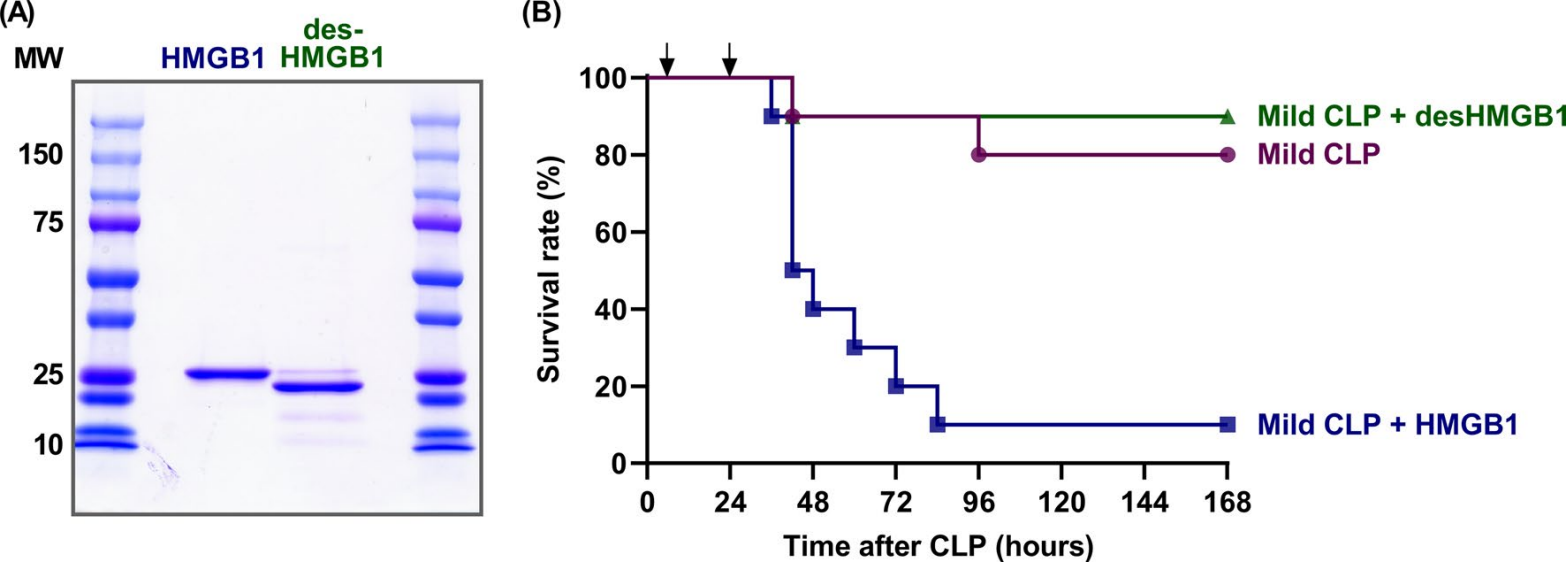
Impact of HMGB1 or desHMGB1 administration on cecal ligation and puncture (CLP)-induced sepsis in mice. **A** Purity of HMGB1 (2 μg/lane) and desHMGB1 (2 μg/lane) was analyzed using sodium dodecyl sulfate-polyacrylamide gel electrophoresis (SDS-PAGE) followed by Coomassie Brilliant Blue (CBB) staining. **B** Male C57BL/6J mice were subjected to a mild model of CLP-induced sepsis. Mice were randomly divided into the following three groups, each containing 10 animals: CLP alone, CLP with HMGB1 administration, and CLP with desHMGB1 administration. Either 2 mg/kg of HMGB1, 2 mg/kg of desHMGB1, or an equal volume of saline was administered into the tail veins of mice at 6 and 24 h after CLP (the time points are indicated by arrows), and the survival rate was monitored for 7 days. The survival rate of the CLP with HMGB1 group was significantly lower than that of the CLP with desHMGB1 group or the CLP alone group (*P* < 0.01, log-rank test)

To clarify the functional differences between HMGB1 and desHMGB1, we first examined the survival of CLP-induced septic mice challenged with purified HMGB1 or desHMGB1. As shown in Fig. 1, the administration of purified HMGB1 significantly reduced the survival rate of CLP mice. In contrast, the administration of purified desHMGB1 had no effect on the survival rate. These findings indicate that HMGB1 and desHMGB1 are very different in terms of their roles in sepsis pathogenesis, and suggest that an analytical method that can discriminate between these two molecules is required for an in-depth understanding of HMGB1-induced pathogenicity.

**Fig. 2:**
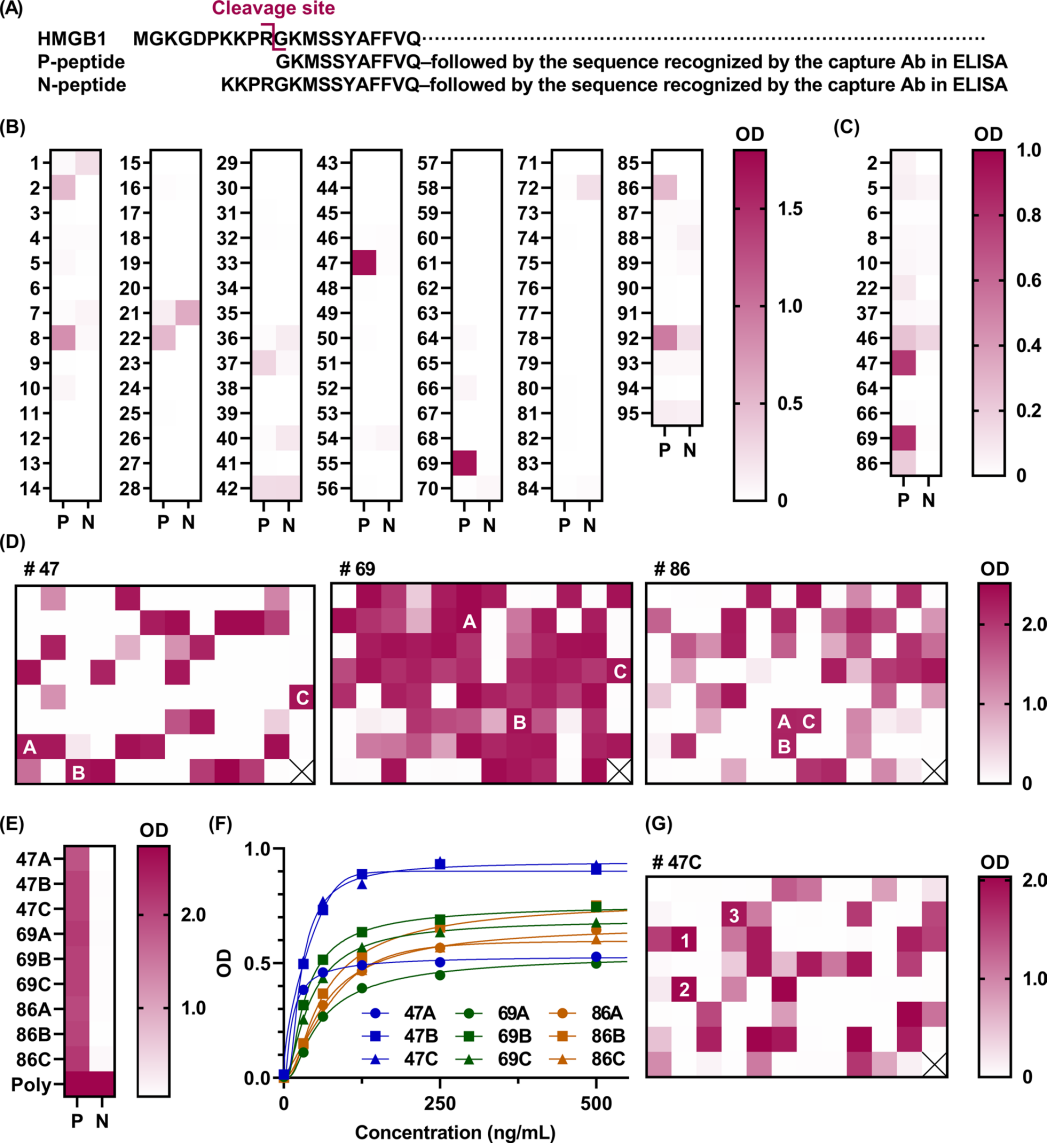
Screening of monoclonal antibodies that recognize desHMGB1 but do not recognize intact HMGB1. **A** The cleavage site of HMGB1, located between amino acid residues R10 and G11, as well as the amino acid sequence of the P-peptide beginning from G11 of HMGB1 and the N-peptide beginning from K7 of HMGB1, are shown. **B**, **C** Of the 3800 hybridoma populations, 95 populations had supernatants that were highly reactive to the desHMGB1 peptide conjugate and underwent the second screening. Microtiter plates were coated with P- or N-peptide and incubated with the supernatants of each hybridoma population (**B**). Alternatively, polystyrene microtiter plates were coated with the capture antibody in the HMGB1 ELISA, incubated with P- or N-peptide, and then incubated with the supernatants of each hybridoma population (**C**). After washing, the plates were incubated with anti-mouse IgG peroxidase-conjugated antibodies, washed again, and incubated with the chromogenic substrate. The optical density (OD) of each well was analyzed and is shown as a heat map. In this screening test, three hybridoma populations (#47, #69, and #86) were specifically reactive to P-peptide but unreactive to N-peptide. **D**, **E** These three hybridoma populations were then each distributed into 95 wells, and the same screening procedure was again conducted to obtain a single hybridoma clone. The results of the antigen (P- or N-peptide)-coated ELISA and the antibody-coated ELISA are shown in **D** and **E**, respectively. **F** The results of the antibody-coated ELISA with different concentrations of desHMGB1 and different types of detection antibody (47A–C, 69A–C, 86 A–C) are shown. **G** We selected the hybridoma clone 47C1, which produced antibodies that specifically recognized desHMGB1

We then attempted to obtain monoclonal antibodies that recognized desHMGB1 but did not recognize intact HMGB1. To this end, we immunized C3H/*lpr* mice with the desHMGB1 peptide conjugate, and isolated spleen and lymph node cells from the immunized donor whose serum showed the highest reactivity to desHMGB1. Ninety-five hybridoma populations with supernatants that were highly reactive to the desHMGB1 peptide conjugate were subjected to the second screening. In this screening test, three hybridoma populations (#47, #69, and #86) were observed to be specifically reactive to the P-peptide, which has a shared N-terminal structure with desHMGB1, but were unreactive to the N-peptide, which has four extra amino acids at the N-terminus (Fig. 2A–C). These three hybridoma populations were then distributed into 95 wells each, and the same screening procedure was again conducted (Fig. 2D–G) to obtain a single hybridoma clone, 47C1, which produced an antibody that selectively recognized desHMGB1.

**Fig. 3:**
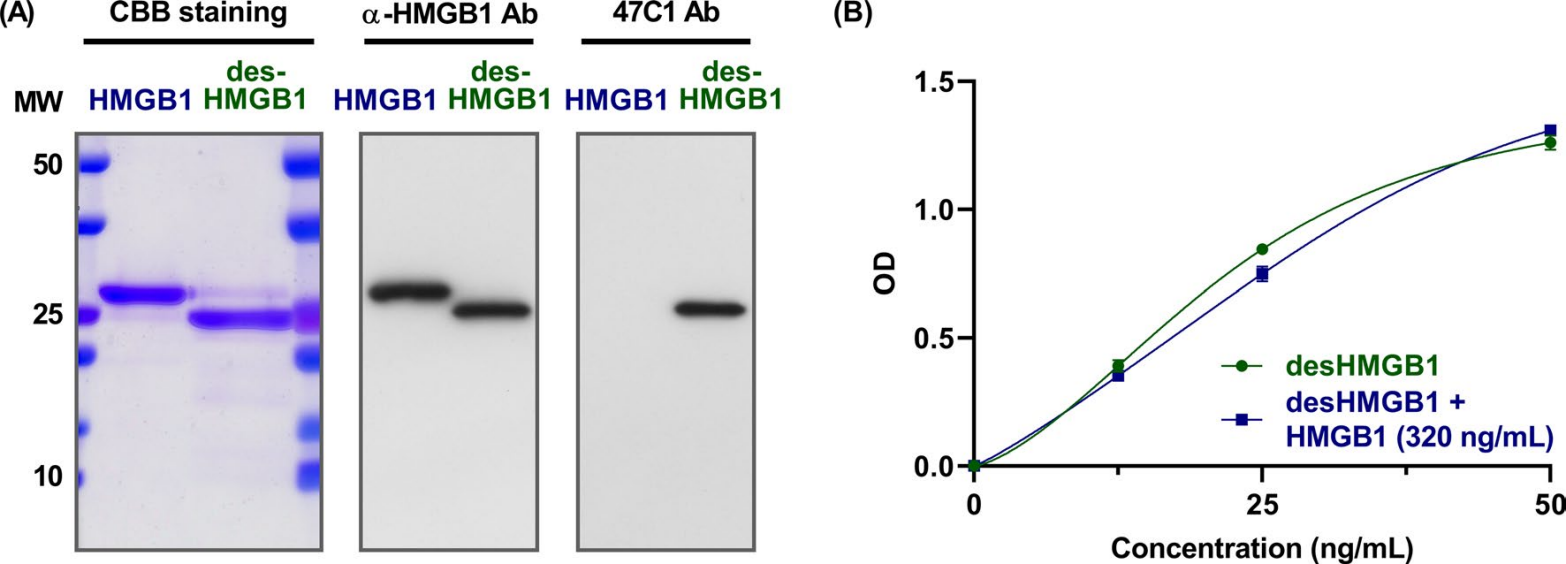
Specificity of the monoclonal antibody 47C1. **A** Purified HMGB1 and desHMGB1 were subjected to SDS-PAGE followed by CBB staining (2 μg protein/lane) or western blotting (50 ng protein/lane). The anti-HMGB1 polyclonal antibody (ab 18256) detected both HMGB1 and desHMGB1, while the anti-desHMGB1 monoclonal antibody 47C1 specifically detected desHMGB1. **B** ELISA for desHMGB1 was constructed as described in “Methods” section. In this assay system, large amounts of intact HMGB1 (320 ng/mL) did not interfere with the detection of desHMGB1 (12.5–50 ng/mL), indicating that the anti-desHMGB1 monoclonal antibody 47C1 did not interact at all with intact HMGB1

To confirm the specificity of the anti-desHMGB1 monoclonal antibody 47C1, we conducted western blot analysis using the 47C1 antibody as well as a conventional anti-HMGB1 polyclonal antibody, which recognizes the carboxy-terminal domain (Lys150 to the carboxy-terminus) of HMGB1. The anti-HMGB1 polyclonal antibody detected both HMGB1 and desHMGB1, while the anti-desHMGB1 monoclonal antibody 47C1 specifically detected desHMGB1 (Fig. 3A). We then constructed an ELISA for desHMGB1 using the 47C1 antibody, as described in “Methods” section. In this assay system, large amounts of intact HMGB1 did not interfere at all with the detection of desHMGB1 (Fig. 3B), further indicating that the anti-desHMGB1 monoclonal antibody 47C1 interacted with desHMGB1 but not with intact HMGB1.

**Fig. 4:**
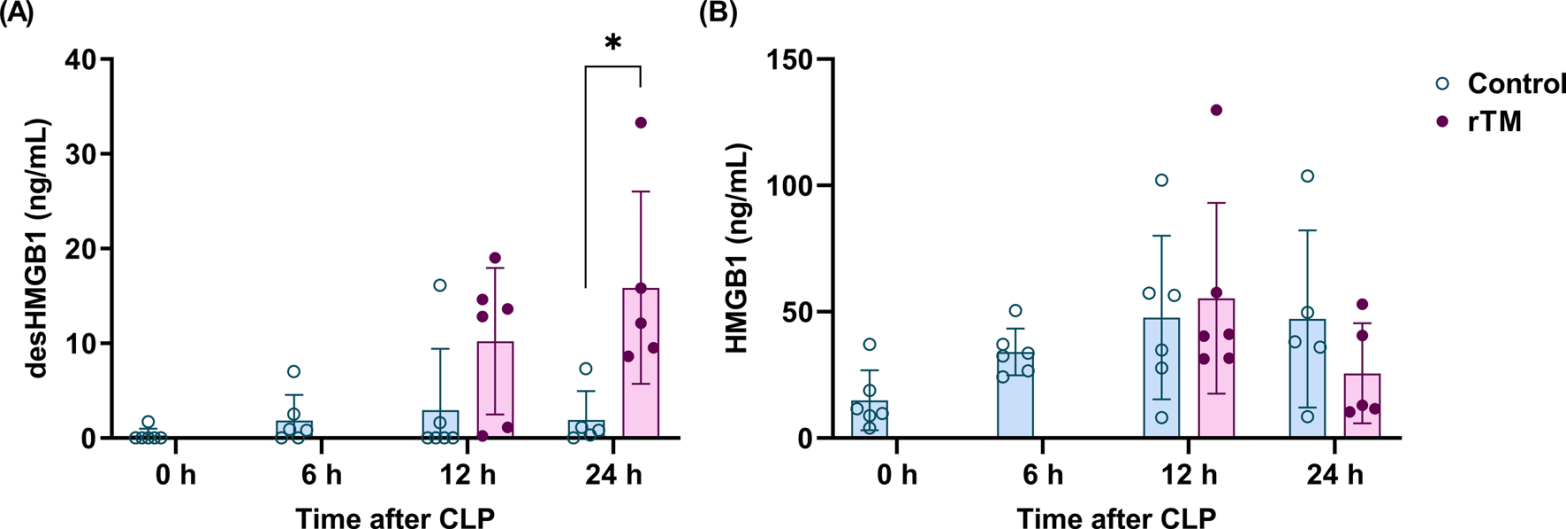
Plasma levels of desHMGB1 in response to rTM treatment in CLP-induced septic mice. Male C57BL/6J mice were subjected to a severe model of CLP-induced sepsis. Mice were randomly divided into the following two groups: those treated with saline and those treated with rTM (5 mg/kg, intravenously) at 6 h after CLP. Plasma samples were collected at 0, 6, 12, and 24 h after CLP, and the plasma levels of desHMGB1 (**A**) and HMGB1 (**B**) were analyzed using ELISA. Six mice were allocated to each time point in each group; however, one mouse in each group became moribund before 24 h. These two mice were sacrificed before 24 h and excluded from further analyses. Data are shown as means ± standard deviations. Statistical significance was determined using the Holm–Šidák method. *P < 0.05

To analyze the potential utility of this new ELISA in evaluating the efficiency of rTM, we examined the plasma desHMGB1 levels of septic mice treated with either saline or rTM. As shown in Fig. 4A, the plasma levels of desHMGB1 at 24 h after CLP were significantly increased in mice treated with rTM compared with those treated with saline. Furthermore, the plasma levels of HMGB1 were not increased by rTM treatment (Fig. 4B). Together, these findings suggest that the measurement of desHMGB1 levels may be an option for evaluating the efficacy of rTM treatment.

## Discussion

In the present study, we obtained monoclonal antibodies that recognized desHMGB1 but did not recognize intact HMGB1. Given that desHMGB1 is a part of intact HMGB1, one might assume that antibodies that interact with desHMGB1 would also interact with intact HMGB1. However, it is possible that the N-terminal edge of desHMGB1 has a unique structure that is not present in intact HMGB1 (Additional file 1: Figure S1); antibodies that interact with this portion may possess a specific affinity for desHMGB1. Indeed, the monoclonal antibody 47C1 interacted with desHMGB1, generated by the proteolytic cleavage of HMGB1, and with the synthetic peptide beginning from Gly11 of HMGB1; however, it did not interact with intact HMGB1 or with the synthetic peptide beginning from Lys7 of HMGB1.

rTM is an anticoagulant drug that is used for the treatment of DIC in Japan; it is also a candidate drug for the treatment of sepsis-associated coagulopathy worldwide (Saito et al. 2007; Vincent et al. 2019; François et al. 2021). The anticoagulant mechanism of rTM is based on its binding to thrombin and the subsequent activation of protein C by the thrombin–rTM complex (Ito et al. 2019). Activated protein C then inactivates coagulation factors Va and VIIIa, thereby inhibiting further thrombin generation (Ito et al. 2020). In addition, the thrombin–rTM complex inactivates HMGB1, thereby suppressing inflammation and neuropathic pain (Ito et al. 2008; Tsujita et al. 2021). To date, laboratory markers that can be used to monitor the efficacy of rTM are lacking, although downstream coagulation markers, such as thrombin–antithrombin complex and d-dimer may be used (Arishima et al. 2018). It is anticipated that desHMGB1 can be used as a direct marker to reflect the efficacy of rTM. However, there are several possible limitations to this approach. Under conditions where excessive thrombin is generated within the vasculature, desHMGB1 can be generated by the thrombin–endogenous thrombomodulin complex even in the absence of rTM. Indeed, desHMGB1 was detected in some of the septic mice who did not receive rTM treatment in this study. Conversely, under conditions where little thrombin is generated within the vasculature, desHMGB1 may not be generated even in the presence of rTM. In this regard, baseline thrombin generation may be an important predictor of rTM treatment responses (Levi et al. 2020). Nevertheless, desHMGB1 was significantly increased in septic mice treated with rTM compared with untreated mice, which indicates that desHMGB1 can be used as a marker of rTM treatment.

The conventional ELISA used worldwide for the measurement of HMGB1 detects both intact HMGB1 and desHMGB1. Given that intact HMGB1 is proinflammatory but desHMGB1 is not, an analytical method that allows the accurate measurement of intact HMGB1 may provide a more in-depth understanding of HMGB1-induced pathogenicity. This can be accomplished by subtracting a desHMGB1 value from a total HMGB1 value. Further studies are needed to clarify the clinical significance of desHMGB1 measurement in managing sepsis and sterile inflammatory diseases.

## Conclusions

In this study, we obtained a desHMGB1-specific monoclonal antibody. ELISA using the novel monoclonal antibody may be an option for the in-depth analysis of HMGB1-induced pathogenicity as well as rTM-mediated therapeutic efficiency.

## Supplementary Information

**Additional file 1: Figure S1.** Theoretical basis of the specific action of the monoclonal antibody 47C1.

## Data Availability

The datasets used and/or analyzed during the current study are available from the corresponding author upon reasonable request.

## Abbreviations

HMGB1: High mobility group box 1 protein
rTM: Recombinant thrombomodulin
DIC: Disseminated intravascular coagulation
NTD: Amino-terminal domain
desHMGB1: HMGB1 degradation product lacking NTD
ELISA: Enzyme-linked immunosorbent assay
SDS-PAGE: Sodium dodecyl sulfate-polyacrylamide gel electrophoresis
BSA: Bovine serum albumin
PBS: Phosphate-buffered saline
CLP: Cecal ligation and puncture

## References

[CR1] Abeyama K, Stern DM, Ito Y, Kawahara K, Yoshimoto Y, Tanaka M, Uchimura T (2005). The N-terminal domain of thrombomodulin sequesters high-mobility group-B1 protein, a novel antiinflammatory mechanism. J Clin Investig.

[CR2] Andersson U, Tracey KJ (2011). HMGB1 is a therapeutic target for sterile inflammation and infection. Annu Rev Immunol.

[CR3] Andrassy M, Volz HC, Igwe JC, Funke B, Eichberger SN, Kaya Z, Buss S (2008). High-mobility group box-1 in ischemia-reperfusion injury of the heart. Circulation.

[CR4] Arishima T, Ito T, Yasuda T, Yashima N, Furubeppu H, Kamikokuryo C, Futatsuki T (2018). Circulating activated protein C levels are not increased in septic patients treated with recombinant human soluble thrombomodulin. Thromb J.

[CR5] François B, Fiancette M, Helms J, Mercier E, Lascarrou JB, Kayanoki T, Tanaka K (2021). Efficacy and safety of human soluble thrombomodulin (ART-123) for treatment of patients in France with sepsis-associated coagulopathy: post hoc analysis of SCARLET [eng]. Ann Intensive Care.

[CR6] Hashimoto T, Ishii J, Kitagawa F, Yamada S, Hattori K, Okumura M, Naruse H (2012). Circulating high-mobility group box 1 and cardiovascular mortality in unstable angina and non-ST-segment elevation myocardial infarction. Atherosclerosis.

[CR7] Hatada T, Wada H, Nobori T, Okabayashi K, Maruyama K, Abe Y, Uemoto S (2005). Plasma concentrations and importance of high mobility group box protein in the prognosis of organ failure in patients with disseminated intravascular coagulation. Thromb Haemost.

[CR8] Ito T, Kawahara K, Okamoto K, Yamada S, Yasuda M, Imaizumi H, Nawa Y (2008). Proteolytic cleavage of high mobility group box 1 protein by thrombin-thrombomodulin complexes. Arterioscler Thromb Vasc Biol.

[CR9] Ito T, Kakihana Y, Maruyama I (2016). Thrombomodulin as an intravascular safeguard against inflammatory and thrombotic diseases. Expert Opin Ther Targets.

[CR10] Ito T, Thachil J, Asakura H, Levy JH, Iba T (2019). Thrombomodulin in disseminated intravascular coagulation and other critical conditions-a multi-faceted anticoagulant protein with therapeutic potential. Crit Care.

[CR11] Ito T, Maruyama I, Shimazaki S, Yamamoto Y, Aikawa N, Hirayama A, Honda G (2020). Effects of thrombomodulin alfa on hemostatic parameters in disseminated intravascular coagulation: post hoc analysis of a phase 3 randomized controlled trial. Res Pract Thromb Haemost.

[CR12] KÖHler G, Milstein C (1975). Continuous cultures of fused cells secreting antibody of predefined specificity. Nature.

[CR13] Levi M, Vincent J-L, Tanaka K, Radford AH, Kayanoki T, Fineberg DA, Hoppensteadt D (2020). Effect of a recombinant human soluble thrombomodulin on baseline coagulation biomarker levels and mortality outcome in patients with sepsis-associated coagulopathy. Crit Care Med.

[CR14] Lotze MT, Tracey KJ (2005). High-mobility group box 1 protein (HMGB1): nuclear weapon in the immune arsenal. Nat Rev Immunol.

[CR15] Rittirsch D, Huber-Lang MS, Flierl MA, Ward PA (2009). Immunodesign of experimental sepsis by cecal ligation and puncture. Nat Protoc.

[CR16] Saito H, Maruyama I, Shimazaki S, Yamamoto Y, Aikawa N, Ohno R, Hirayama A (2007). Efficacy and safety of recombinant human soluble thrombomodulin (ART-123) in disseminated intravascular coagulation: results of a phase III, randomized, double-blind clinical trial. J Thromb Haemost.

[CR17] Sanders C (1977). A method for the fractionation of the high-mobility-group non-histone chromosomal proteins. Biochem Biophys Res Commun.

[CR18] Singer M, Deutschman CS, Seymour CW, Shankar-Hari M, Annane D, Bauer M, Bellomo R (2016). The third international consensus definitions for sepsis and septic shock (Sepsis-3). JAMA.

[CR19] Taniguchi N, Kawahara K, Yone K, Hashiguchi T, Yamakuchi M, Goto M, Inoue K (2003). High mobility group box chromosomal protein 1 plays a role in the pathogenesis of rheumatoid arthritis as a novel cytokine. Arthritis Rheum.

[CR20] Tong HS, Tang YQ, Chen Y, Qiu JM, Wen Q, Su L (2011). Early elevated HMGB1 level predicting the outcome in exertional heatstroke. J Trauma.

[CR21] Tsujita R, Tsubota M, Sekiguchi F, Kawabata A (2021). Role of high-mobility group box 1 and its modulation by thrombomodulin/thrombin axis in neuropathic and inflammatory pain. Br J Pharmacol.

[CR22] Vincent JL, Francois B, Zabolotskikh I, Daga MK, Lascarrou JB, Kirov MY, Pettila V (2019). Effect of a recombinant human soluble thrombomodulin on mortality in patients with sepsis-associated coagulopathy: the SCARLET randomized clinical trial. JAMA.

[CR23] Volz HC, Laohachewin D, Schellberg D, Wienbrandt AR, Nelles M, Zugck C, Kaya Z (2012). HMGB1 is an independent predictor of death and heart transplantation in heart failure. Clin Res Cardiol.

[CR24] Wang H, Bloom O, Zhang M, Vishnubhakat JM, Ombrellino M, Che J, Frazier A (1999). HMG-1 as a late mediator of endotoxin lethality in mice. Science.

[CR25] Wang H, Yang H, Czura CJ, Sama AE, Tracey KJ (2001). HMGB1 as a late mediator of lethal systemic inflammation. Am J Respir Crit Care Med.

[CR26] Xue J, Suarez JS, Minaai M, Li S, Gaudino G, Pass HI, Carbone M (2021). HMGB1 as a therapeutic target in disease. J Cell Physiol.

[CR27] Yamamoto T, Ono T, Ito T, Yamanoi A, Maruyama I, Tanaka T (2010). Hemoperfusion with a high-mobility group box 1 adsorption column can prevent the occurrence of hepatic ischemia-reperfusion injury in rats. Crit Care Med.

[CR28] Yang H, Ochani M, Li J, Qiang X, Tanovic M, Harris HE, Susarla SM (2004). Reversing established sepsis with antagonists of endogenous high-mobility group box 1. Proc Natl Acad Sci USA.

