# Supplementary figures and images for: Specific detection of high mobility group box 1 degradation product with a novel ELISA
Source: Mol Med. 2021 Jun 9;27:59. doi: 10.1186/s10020-021-00323-1 (PMC8190996; doi:10.1186/s10020-021-00323-1)

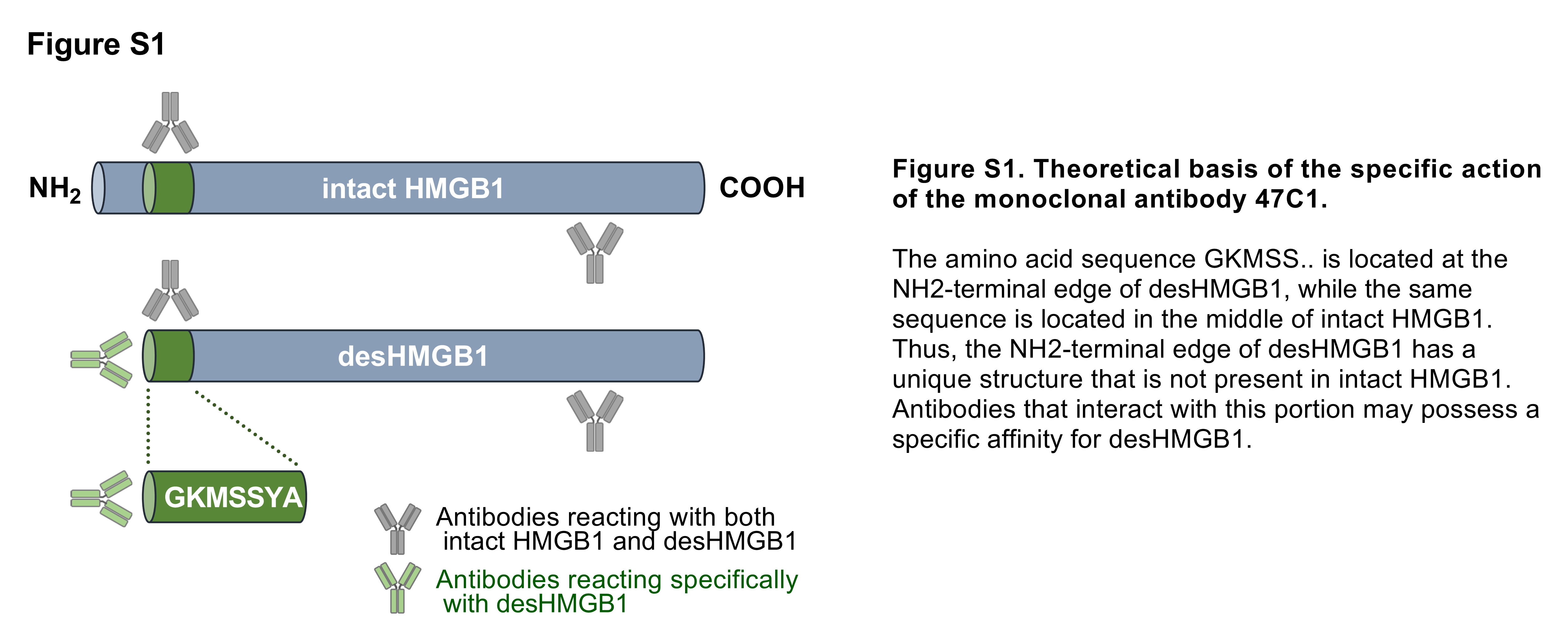

Supplement: Supplementary file 1 — Additional file 1: Figure S1. Theoretical basis of the specific action of the monoclonal antibody 47C1. [file 10020_2021_323_MOESM1_ESM.tif]
